# Effect of Arbuscular Mycorrhizal Fungi on *Pratylenchus penetrans* Infestation in Apple Seedlings under Greenhouse Conditions

**DOI:** 10.3390/pathogens7040076

**Published:** 2018-09-21

**Authors:** An Ceustermans, Wendy Van Hemelrijck, Jelle Van Campenhout, Dany Bylemans

**Affiliations:** 1 Research Station for Fruit Cultivation, B-3800 Sint-Truiden, Belgium; wendy.vanhemelrijck@pcfruit.be (W.V.H.); jelle.vancampenhout@pcfruit.be (J.V.C.); dany.bylemans@pcfruit.be (D.B.); 2 Department of Biosystems, KU Leuven, B-3001 Heverlee, Belgium

**Keywords:** Arbuscular Mycorrhizal Fungi, replant disease, apple seedlings, *Pratylenchus penetrans*

## Abstract

A major problem in fruit cultivation in Flanders is replant disease due to a lack of uncultivated soils available for new plantings. Replant disease can cause poor growth and affect time to full production, however Arbuscular Mycorrhizal Fungi (AMF) can prove their usefulness with regard to these problems. To further investigate the effect of AMF on nematodes, different AMF species were amended to potted apple seedlings in the presence of the nematode *Pratylenchus penetrans*. Generally, apple seedlings grew better in the presence of nematodes when mycorrhiza were inoculated into the soil. Moreover, a positive correlation (R^2^ ≥ 0.88) was found between the percentage root length colonization of the roots of apple seedlings, by AMF species, and nematode reduction in the soil of the seedlings. Indigenous AMF could colonize the roots of apple seedlings the most efficiently, resulting in a higher biocontrol effect. Besides, a synergistic effect was observed when two AMF strains were applied together leading to a significant growth response of the seedlings.

## 1. Introduction

A main problem in the cultivation of apple in Flanders is the limited presence of pristine soils for new plantations. Hence, fruit growers do not have other options than to repeatedly use soil with the same history. This can lead to replant disease, which is common to all major apple growing regions of the world [1]. Symptoms of replant disease include a reduced growth of trees, discolored roots, root tip necrosis, reduction in root biomass, delay in fruit production, shortened internodes, and a general reduction in overall fruit yield and quality [1]. Both biotic and abiotic factors can lead to this problem, but a complex of nematodes and fungi (especially *Pythium* sp., *Phytophthora* sp. and *Pratylenchus* sp.) is the most important cause [2,3,4,5,6]. Especially root lesion nematodes, like *Pratylenchus penetrans*, have been closely associated with replant disease of cherries, apple and peaches [7,8]. Until recently, this problem was resolved by the use of chemical soil disinfestation. With a prohibition on the use of most of these products, the search for alternatives is nowadays an absolute necessity. The current alternative solutions (sowing of Rye grass, Tagetes, Japanese Oat or Phacelia) require a lot of time (one year extra compared to chemical disinfestation) between grubbing of the trees and the new plantation. Moreover, these green manures can form an attraction pole for other diseases (e.g., nematodes). This was observed for Tagetes during field trials executed at the experimental garden for pome and stone fruits of our research center (pcfruit npo) [9]. A more sustainable solution could be the use of Arbuscular Mycorrhizal Fungi (AMF), which are naturally present in the soil. AMF form a symbiosis with the roots of 80% of all plant species and are abundantly present in the soil of most ecosystems [10]. Moreover, AMF are also naturally present in the roots of apple trees and species that belong to the genera of *Glomus*, *Claroideoglomus*, *Paraglomus*, *Scuttelospora*, *Diversispora,* and *Acaulospora*. This was observed during a survey of 24 apple orchards (among which organic, conventional and standard orchards and nurseries) in the Flemish region of Belgium [11]. In general, AMF form a vast mycelial growth around the rhizodermis and in the root cells. They enlarge the contact surface and increase the storage capacity of water and nutrients by the presence of millions of small vesicules and arbuscules [12]. AMF can also cause multiple changes in root morphology, like increasing the number of adventitious and lateral roots, and their colonization usually results in greater branching [13]. In addition, mycorrhization of plants with AMF can cause a significant mobilization of insoluble phosphorus in the soil and uptake by plants [14]. Consequently, a tree can possess more vigor through the additional uptake of nutrients from the soil. As a result, fewer fertilizers have to be used [15] and young plants or trees can reach their maximal production capacity one year earlier. Indeed, different research demonstrated that AMF can have a positive effect on the growth and vitality of several plant species, among fruit trees [16,17,18,19,20,21,22,23]. Furthermore, it was shown that AMF can reduce infections with soil pathogens, like *Erwinia carotovora*, *Fusarium oxysporum*, *Pseudomonas syringae*, *Phytophthora* spp., and *Alternaria solani* [24,25,26,27,28,29]. Additionally, plant parasitic nematodes, like *Meloidogyne* spp., *Pratylenchus penetrans*, and *Nacobbus aberrans* were reduced after inoculation with AMF in tomato, dune grass, olive, and carrot [30,31,32,33,34]. However, the effect of AMF on nematode reduction is apparently ambiguous with fruit trees. Mycorrhizal inoculation had no effect on the number of nematodes with rootstocks of cherry, quince, and plum [35,36,37,38], while a lower amount of nematodes was shown with apple, peach, and pear [39,40,41,42,43]. In addition, most of the commercial mycorrhiza products contain only generally occurring species, which may not be the most efficient for fruit trees. Therefore, the influence of a mix of different AMF species, originating from apple roots themselves (containing general as well as host-specific species), on the growth of apple seedlings in the presence of the nematode *P. penetrans* was evaluated under greenhouse conditions to further investigate the effect of AMF on nematodes. Besides, two other non-host-specific AMF species, i.e., *Acaulospora longula* and *Claroideoglomus claroideum*, were included as their efficiency against other nematodes in other plant species has already been described in literature [44,45]. Moreover, these AMF species are easy to cultivate. The aims of this study were to determine if mycorrhized apple seedlings can grow better in the presence of nematodes and if AMF plays a role in the reduction of nematode levels.

## 2. Results

Apple seedlings, which had to cope with the nematode *P. penetrans,* showed a reduced root system compared to the control plants. As a result, a reduced growth of the seedlings was observed (Table 1). However, apple seedlings grew better in presence of nematodes when mycorrhiza were inoculated in the soil. Most of these seedlings grew even better compared to apple seedlings where no nematodes or AMF were added to the soil (control). The highest growth response was obtained for seedlings that were inoculated with a combination of two AMF species, i.e., *C. claroideum* and *A. longula*. Furthermore, seedlings inoculated with this combination had a significantly higher shoot length and fresh weight of the roots compared to the seedlings inoculated with only one of both species. Additionally, seedlings inoculated with *Glomus intraradices* and nematodes showed a significantly better growth compared to seedlings, which were only subjected to nematodes. No difference in growth was found between seedlings inoculated with only one general AMF species (*G. intraradices*) and seedlings inoculated with a mix of general and host-specific AMF species.

Colonization with AMF was observed in the roots of non-inoculated seedlings, despite the soil being sterilized (Table 2). However, the AMF colonization degree was much higher for seedlings inoculated with different AMF species compared to seedlings without AMF inoculation. The highest AMF root length colonization was detected in the roots of seedlings inoculated with the indigenous AMF mix. This was significantly higher than in the roots of seedlings inoculated with the combination of *C. claroideum* and *A. longula* or inoculated with a single AMF species. The same trend was observed for the vesicular and arbuscular colonization degree.

The number of nematodes in the soil was reduced by at least 50% for seedlings inoculated with AMF, as compared to the seedlings only inoculated with nematodes, except for seedlings inoculated with *C. claroideum* in which a small increase in nematode numbers was observed (Table 2). Seedlings inoculated with the indigenous AMF mix showed the highest nematode reduction in the soil. A positive correlation (R^2^ ≥ 0.88) was found between percentage root length colonization, percentage arbuscular colonization or percentage vesicular colonization of the roots of apple seedlings by efficient AMF species and nematode reduction in the soil of the seedlings.

## 3. Discussion

Our results revealed that apple seedlings, subjected to the nematode *P. penetrans* have a reduced growth in the absence of inoculated mycorrhiza, as compared to seedlings growing in a nematode and mycorrhiza free substrate. However, this reduced growth was not significant despite the number of added nematodes to the seedlings corresponded to the economic threshold. The economic threshold is based on an initial soil population level that will multiply over the growing season and which causes economic damage to the crop later in that particular season [46]. Still, the final soil population level in our trial was below this threshold (100 nematodes per 100 g of soil), indicating that seedling growth conditions or time were not very favorable to allow a significant expansion of the nematode population. A threefold increase in nematode level was counted, for example, after a period of 2.5 years in pots of 10 L during semi-practical field trials. Nonetheless, these seedlings had also a reduced root system compared to seedlings growing in absence of nematodes. The same observation of reduced root growth was noticed for another group of seedlings (treatment: *C. claroideum* + *Pp*), where the same nematode level was counted. Root-lesion nematodes, like *P. penetrans*, cause degradation of cells in the epidermis and cortex of underground plant organs. These activities reduce the amount of root branching and the ability of roots to absorb water and nutrients [47] and is in concordance with our study. It must be noted that the number of nematodes was only counted in the soil of the seedlings and not in the roots. However, the proportion of the number of nematodes per pot compared to the number of nematodes per root system remained the same in the study of Forge et al. [43].

Various beneficial effects were observed when different AMF species were added to the soil of the seedlings growing in a substrate contaminated with nematodes. The growth of all the AMF-inoculated seedlings coping with nematodes was comparable to or even better than the seedlings growing in a nematode and mycorrhiza free substrate, indicating that the seedlings were able to withstand the damage caused by the nematodes. Different mechanisms can be involved in the biocontrol effect of AMF against plant-parasitic nematodes that include enhanced plant tolerance (by a higher indirect nutrient uptake or by an altered root morphology which facilitates direct nutrient uptake), direct competition for nutrients and space, induced systemic resistance, and altered rhizosphere interactions [48]. Moreover, biocontrol probably results from a combination of different mechanisms [48]. While this was not the scope of our study, we hypothesize that it could indeed be a combination of enhanced plant tolerance and competition for space. We noticed an increased fresh weight of the roots of the AMF-inoculated seedlings compared to non-AMF-inoculated seedlings all coping with nematodes. All the AMF species that were included in our study showed this effect except *C. claroideum*. It has been shown in other studies that mycorrhizal plants often show increased root growth and branching [48]. In the study of Hosseini et al. [49], the fresh weight of the roots of apple rootstocks was significantly increased after inoculation with *C. etunicatum*, *G*. *versiforme*, or *Rhizophagus intraradices*. The fresh root weight of peach seedlings was also significantly increased after inoculation with *G. mosseae* or *G. versivorme* compared with the non-AMF control [50]. Moreover, increased root branching observed in mycorrhizal plants has been suggested to have implications for pathogen infection and might even counterbalance the suppressed root growth caused by plant-parasitic nematodes [48], which is in agreement with our study. As a consequence, the direct absorption of nutrients is facilitated in AMF-inoculated plants by the improvement of the structure of the root system [51]. It must be noted that the AMF species *C. claroideum* could not counterbalance the suppressed root growth under the experimental conditions, but still caused a higher shoot length and stem diameter of the apple seedlings compared to seedlings with the same amount of nematodes but without inoculated AMF. As both groups of seedlings had a reduced root system compared to seedlings without nematodes, *C. claroideum* offered another benefit to the plants, which could enhance plant tolerance by a higher indirect nutrient uptake. Other researchers showed that inoculation of apple rootstocks with another species of *Claroideoglomus*, i.e., *C. etunicatum*, significantly increased leaf concentrations of N, P, Ca, Mg, and Zn, compared to the control plants [49]. Another possible mechanism of biocontrol is competition for space, which implies that a higher AMF colonization degree of the root should lead to a higher level of biocontrol [48]. Indeed, a positive correlation was found between percentage root length colonization, percentage arbuscular colonization, or percentage vesicular colonization of the roots of the apple seedlings by efficient AMF species and nematode reduction in the soil of the seedlings in our study. However, this positive correlation has to be carefully interpreted as it is only based on four data points. Moreover, this correlation did not apply for all the tested AMF species. The percentage root length colonization of *C. claroideum* was comparable to *A. longula* or their combination, but did not result in a reduction of nematodes. We did not find a proper explanation for this observation within our trial set-up. On the contrary, we found no correlation between root colonization by AMF and growth response of the seedlings. These findings are confirmed by the research of Forge et al. [43], whereby differences in the overall growth promotion of the *Glomus* species they evaluated were not clearly related to differences in root colonization. According to Forge et al. [43], colonization may not be the best indicator of mycorrhiza-enhanced nutrient uptake and overall growth, but could be a good indicator of pathogen suppression.

The reduction of the nematode population in the soil of the AMF-inoculated seedlings was only significant for the mix of indigenous AMF species. This could be explained by the high variation in number of nematodes between replications. Nevertheless, indigenous AMF colonized the roots of apple seedlings the most efficient, in our study, resulting in the highest biocontrol effect. When the number of nematodes would exceed the economic threshold level, this higher biocontrol effect of indigenous AMF might also lead to a more significant growth response of the seedlings. Such a higher efficiency of indigenous AMF strains has been previously revealed in several other studies, as reviewed by Berruti et al. [52]. Root infection and the multiplication of the root-feeding nematode *P. penetrans* were significantly reduced by a native inoculum of AMF in dune grass [32]. Affokpon et al. [44] reported that native strains were superior in suppressing root-knot nematodes compared to commercial products. Additionally, Séry et al. [53] observed that mycorrhizal cassava plants were either resistant or tolerant to root-knot nematodes when native strains were used.

A significant growth response was observed when either *G. intraradices* or a mix of indigenous AMF were added to the seedlings. *Glomus intraradices* is a globally distributed AMF species present in both natural and anthropogenic habitats [54]. It can colonize a large variety of host plants, survive long-term storage and can be easily and massively propagated [52]. This AMF species is therefore often used in commercial AMF products, e.g., MYC4000, the product which was incorporated in our trial. This AMF species, was probably also present in the mix of indigenous species, as it was the most common AMF taxon in our previous observational studies occurring in almost all samples [55]. As nematode reduction was higher in the soil containing the mix of indigenous species, also other AMF species besides *G. intraradices* might have a biocontrol effect. No significant differences were found in the growth response of seedlings inoculated with *G. intraradices* or with the mix of different *Glomus* and *Claroideoglomus* species, despite a further nematode reduction from 68 to 97 percent, respectively. The additional growth response of these groups of seedlings might be attributed to the presence of *G. intraradices*. Other authors reported that peach rootstocks, inoculated with *G. intraradices,* were significantly taller and had significantly thicker diameters than non-inoculated plants [56].

The highest growth response of seedlings (as well as shoot as roots) was observed when two AMF species, i.e., *C. claroideum* and *A. longula,* were inoculated together in the soil around the apple seedlings. This was significantly higher as compared to seedlings inoculated with only one of these species. This cannot solely be explained by a higher nematode reduction as there was no difference in the number of nematodes for the seedlings inoculated with both AMF species and seedlings containing only *A. longula*. Rather, a synergistic effect was observed whereby *A. longula* was probably responsible for a higher fresh root weight and the nematode reduction and *C. claroideum* for a higher indirect nutrient uptake, together resulting in a significant growth response of the seedlings. Such a synergistic effect of dual inoculation of arbuscular mycorrhiza compared to a single species inoculum that was previously reported by Khan et al. [57] and led in their study to a better growth and nutrient uptake of *Medicago sativa*. Additionally, the height of citrus seedlings was significantly higher after mycorrhizal interaction treatments compared to single mycorrhizal inoculation [58]. In addition, Chen et al. [59] observed that a mix of AMF spp. from different genera, like in our study, had a better effect on cucumber growth as compared to a single AMF sp. or a mix of AMF spp. from the same genus. Our results are also in agreement with Alarcón et al. [60] who hypothesized that the sum of interrelationships among AMF species are mutually complemented and result in greater benefits on the growth of seedlings of papaya. No significant differences were observed compared to the control when they inoculated the AMF species (among which *C. claroideum*) separately to papaya plants, like in our study.

The sequence of inoculation has also been suggested as an important factor in mycorrhizal biocontrol [61]. It has been hypothesized that the AMF inoculation has to be prior to the inoculation with pathogens to obtain an effective biocontrol effect [61]. However, in our trial, AMF and nematodes were inoculated at the same time resulting in a significant biocontrol effect when native AMF strains were used. These results are in agreement with Alban et al. [62] who co-inoculated native AMF strains with *Meloidogyne exigua* in coffee plants, which lead to a low to medium nematode infestation rate. Additionally, de la Peña et al. [32] showed that the number of *P. penetrans* was significantly reduced in roots and soil when native AMF strains and nematodes were inoculated simultaneously in dune grass. A possible consequence of simultaneous inoculation of AMF and nematodes under circumstances of competition for nutrients and space, is a negative effect of these nematodes on AMF colonization of the roots [48]. In our study, this negative effect of co-inoculation was not observed since the AMF colonization of the roots was, in general, higher compared to another preliminary trial with apple seedlings in the absence of nematodes, even though a lower amount of inoculated AMF spores (220 compared to 300) was used (data not shown).

## 4. Materials and Methods

### 4.1. Arbuscular Mycorrhizal Fungi and Nematodes

Two non-host-specific AMF species, i.e., *Acaulospora longula* (BEG ID 8) and *Claroideoglomus claroideum* (BEG ID 31) were purchased at the International Bank for the Glomeromycota (www.i-beg.eu). Both species originate from grasses and were cultured in a sterile mix of potting soil and quartz sand with grass (Sorghum) as a host plant during at least six months. A commercial AMF product, MYC 4000, containing spores of *Glomus intraradices*, was also included in the trial. To investigate the effect of indigenous AMF species (as well as generalists as host-specific ones), roots and soil were gathered in an organic apple orchard (50°55′29″ N, 4°56′27″ E) and stored in a cooled room (4 °C) until trial set-up. Afterwards, the roots were cut into fragments smaller than 1 cm and mixed with the soil and tap water (10:1). The mixture contained 13 species belonging to the Glomeraceae and Claroideoglomeraceae families based on 454-pyrosequensing.

The nematode suspension containing *Pratylenchus penetrans* was provided by Wageningen Plant Research.

### 4.2. Plant Material and Soil

Apple seedlings were grown from seeds (*Malus domestica* cv. Golden delicious) originating from orchards belonging to the research center Pcfruit npo. Firstly, seeds were submitted to cold stratification by laying the seeds in plastic boxes filled with potting soil placed at a temperature of 4 °C during at least 2 months to obtain a better germination afterwards. Germination of the seeds took place in a climate chamber under controlled environmental conditions (14/10 h light/dark regime, 20/15 °C and 75%/90% relative humidity) during 2 weeks. Afterwards, seedlings were transplanted in potting soil and were grown further in the greenhouse until trial set-up (21.7 ± 2.6 °C; 40.2 ± 8.4% RV; 20.0 klux).

The soil used in the trial also originates from an orchard belonging to the research center Pcfruit npo (50°46′24″ N, 5°9′38″ E) to mimic practical circumstances. The soil was a light loam with 0.89% organic matter and a pH of 6.6. Concentrations of P, K, Mg, Ca, and Na were 22, 17, 18, 195, and 1.3 mg per 100 g of soil, respectively, with an available P content of 89.4 mg per kg dry matter. The soil was autoclaved during 20 min at 121 °C at 2 bar (Fedegari autoclave 4451E) just before trial set-up.

### 4.3. Trial Set-Up

Apple seedlings of approximately 4 weeks old were transplanted in 1 L pots with a mixture of sterilized loam soil and quartz sand (proportion 1:1). All seedlings received 1 g of a slow release fertilizer (Substral Osmocote^®^ NPK(Mg): 17-9-11(-2)) at the beginning of the trial. The trial was carried out in a greenhouse during 6 months (May until October 2016) (22.6 ± 3.6 °C; 56.0 ± 14.2% RV; 20.0 klux). Seven treatments were performed: (1) Seedlings in absence of nematodes or AMF, (2) seedlings inoculated with *P. penetrans*, (3) seedlings inoculated with *P. penetrans* and *C. claroideum*, (4) seedlings inoculated with *P. penetrans* and *A. longula*, (5) seedlings inoculated with *P. penetrans* and *G. intraradices*, (6) seedlings inoculated with *P. penetrans* and a mix of 13 indigenous AMF species, and (7) seedlings inoculated with *P. penetrans*, *C. claroideum* and *A. longula*. A certain amount of substrate containing 220 AMF spores were added around the roots of each AMF inoculated seedling. The efficiency of this method was already shown with several plant species in other research [58,62,63]. Hundreds of nematodes per 100 g of substrate were added to each seedling inoculated with *P. penetrans* by pouring the nematode solution into 4 small holes of 3 cm deep and 2 cm away from the stem of the seedlings. Growth parameters, i.e., shoot height, stem diameter, and number of leaves were measured every month. Fresh weight of the roots was measured at the end of the experiment (after 6 months) and mycorrhizal colonization in the roots was determined for 5 replicates of each treatment with the gridline intersection method [64]. The number of *P. penetrans* was counted in 3 subsamples using a modification of the Baermann funnel method [65] after the soil of each seedling was mixed per treatment.

### 4.4. Statistical Analysis

Statistical analysis was executed with the aid of the software Unistat 6.5. Bartlett box F test was used to test the hypothesis of homogeneity of variance. If this hypothesis was met, data were analyzed by the analysis of variance (ANOVA). Duncan’s test was applied for multiple comparisons of group means. Otherwise, the non-parametric Friedman test was utilized to detect differences between treatments.

## 5. Conclusions

AMF can offer a sustainable solution for replant disease in apple as they offer apple seedlings protection against nematodes and can provide extra nutrients to the seedlings. Root colonization and functionality, however, differed between AMF strains. Indigenous AMF could colonize the roots of apple seedlings the most efficiently, resulting in a higher biocontrol effect. Moreover, a synergistic effect was observed when two AMF strains were applied together leading to a significant growth response of the seedlings. Future research should incorporate the determination of which AMF species of the indigenous mix provide the biocontrol effect. Afterwards, the most efficient AMF species should be tested in trials with the common rootstock of apple (i.e., M9) and later in field trials in replant situations.

## Figures and Tables

**Table 1:** The effect of single, dual, multiple (mix of 13 indigenous mycorrhiza species) species and a commercial AMF product on the increase in shoot height, increase in stem diameter, number of leaves, and fresh weight of the roots of apple seedlings grown in the presence of the nematode *Pratylenchus penetrans* (*Pp*) measured 6 months after trial set-up.

Treatment	Increase in Shoot Height (cm)	Increase in Stem Diameter (mm)	Number of Leaves	Fresh Weight of Roots (g)
Control	43.5 ± 3.9 ab	3.05 ± 0.23 ab	32 ± 3 a	2.47 ± 0.30 ab
*Pratylenchus penetrans* (*Pp*)	38.3 ± 2.6 a	2.80 ± 0.16 a	32 ± 3 a	1.81 ± 0.22 a
*Claroideoglomus claroideum* + *Pp*	42.4 ± 3.5 ab	3.24 ± 0.17 abc	32 ± 4 a	1.85 ± 0.25 a
*Acaulospora longula* +*Pp*	44.7 ± 5.9 ab	3.02 ± 0.20 ab	37 ± 2 ab	2.51 ± 0.25 ab
*Glomus intraradices* + *Pp*	52.9 ± 3.5 bc	3.42 ± 0.12 bc	42 ± 2 b	3.28 ± 0.32 bc
Mix of indigenous species + *Pp*	53.9 ± 3.3 bc	3.31 ± 0.09 abc	39 ± 1 ab	2.92 ± 0.40 ab
*C. claroideum* + *A. longula* + *Pp*	60.7 ± 4.0 c	3.73 ± 0.16 c	44 ± 1 b	4.80 ± 1.02 c

Data are means of 16 replicates ± standard error. Columns with different letters are significantly different (*p* < 0.05).

**Table 2:** Percentage of root length colonization, arbuscular colonization, vesicular colonization in the roots and nematode reduction near the roots of apple seedlings after inoculation with single, dual, multiple (mix of 13 indigenous mycorrhiza species) species, and a commercial product.

**Treatment**	**Root Length Colonization (%)**	**Arbuscular Colonization (%)**	**Vesicular Colonization (%)**	**Nematode Reduction (%)**
Control	1.85 ± 1.76 a	0.77 ± 0.77 a	0.23 ± 0.23 a	-
*Pratylenchus penetrans* (*Pp*)	0.49 ± 0.49 a	0.00 ± 0.00 a	0.00 ± 0.00 a	0 a
*Claroideoglomus claroideum* + *Pp*	14.68 ± 6.92 ab	5.43 ± 2.10 ab	0.75 ± 0.67 a	-8 a
*Acaulospora longula* + *Pp*	14.81 ± 4.61 ab	4.80 ± 1.58 ab	0.45 ± 0.30 a	50 ab
*Glomus intraradices* + *Pp*	19.54 ± 5.48 b	6.13 ± 2.15 ab	0.53 ± 0.43 a	68 ab
Mix of indigenous species + *Pp*	37.01 ± 9.32 c	9.77 ± 2.79 b	6.66 ± 2.38 b	97 b
*C. claroideum* + *A. longula* + *Pp*	11.90 ± 2.57 ab	4.96 ± 1.42 ab	0.00 ± 0.00 a	54 ab

Data are means of 5 replicates ± standard error, except for NR% where *n* = 3. Columns with different letters are significantly different (*p* < 0.05).
